# Benefits of subsidence control for coastal flooding in China

**DOI:** 10.1038/s41467-022-34525-w

**Published:** 2022-11-14

**Authors:** Jiayi Fang, Robert J. Nicholls, Sally Brown, Daniel Lincke, Jochen Hinkel, Athanasios T. Vafeidis, Shiqiang Du, Qing Zhao, Min Liu, Peijun Shi

**Affiliations:** 1 Institute of Remote Sensing and Earth Sciences, Hangzhou Normal University, Hangzhou, 311121 China; 2 Zhejiang Provincial Key Laboratory of Urban Wetlands and Regional Change, Hangzhou, 311121 China; 3 Academy of Disaster Reduction and Emergency Management, Ministry of Emergency Management & Ministry of Education, Beijing, 100875 China; 4 School of Geographic Sciences, East China Normal University, Shanghai, 200241 China; 5 School of Engineering, University of Southampton, Southampton, SO16 7QF UK; 6 Tyndall Centre for Climate Change Research, University of East Anglia, Norwich Research Park, Norwich, NR4 7TJ UK; 7 Global Climate Forum e.V. (GCF), Berlin, 10178 Germany; 8 Division of Resource Economics, Albrecht Daniel Thaer-Institute and Berlin Workshop in Institutional Analysis of Social-Ecological Systems (WINS), Humboldt-University, Berlin, 10099 Germany; 9 Coastal Risks and Sea-Level Rise Research Group, Department of Geography, Christian-Albrechts-University Kiel, Kiel, 24098 Germany; 10 School of Environmental and Geographical Sciences, Shanghai Normal University, Shanghai, 200234 China; 11 State Key Laboratory of Earth Surface Processes and Resource Ecology (ESPRE), Beijing Normal University, Beijing, 100875 China

**Keywords:** Climate-change mitigation, Governance

## Abstract

Land subsidence is impacting large populations in coastal Asia via relative sea-level rise (RSLR). Here we assesses these risks and possible response strategies for China, including estimates of present rates of RSLR, flood exposure and risk to 2050. In 2015, each Chinese coastal resident experienced on average RSLR of 11 to 20 mm/yr. This is 3 to 5 times higher than climate-induced SLR, reflecting that people are concentrated in subsiding locations. In 2050, assuming these subsidence rates continue, land area, population and assets exposed to the 100-year coastal flood event is 20%-39%, 17%-37% and 18%-39% higher than assuming climate change alone, respectively. Realistic subsidence control measures can avoid up to two thirds of this additional growth in exposure, with adaptation required to address the residual. This analysis emphasizes subsidence as a RSLR hazard in China that requires a broad-scale policy response, utilizing subsidence control combined with coastal adaptation.

## Abstract (web summary)

Chinese coastal populations are concentrated in subsiding locations, and also subject to sea-level rise. Here the authors find that more areas, population and assets are exposed to coastal flooding by 2050 but realistic subsidence control measures can avoid additional risks.

## Introduction

Coastal areas are threatened by increased flooding during the 21st century and beyond due to climate-induced sea-level rise (SLR)^1^. However, the impact of human-induced subsidence^2^ and its effect on relative sea-level rise (RSLR)^3^ at broad scales has been less assessed. RSLR comprises the sum of climate-driven SLR, glacial isostatic adjustment (GIA), tectonic vertical land movements, natural subsidence due to consolidation and human-induced subsidence due to ground fluid withdrawal, drainage and other causes^3–5^. All these processes need to be considered in coastal risk and adaptation assessments. A number of important coastal cities built completely or partly on deltaic deposits, such as New Orleans, Bangkok, Jakarta, Shanghai and Tokyo, have already experienced several metres of human-induced subsidence through the 20th/early 21st century. Most of these cities continue to subside today^6,7^. Hence, subsidence is a process with major consequences for flood risk and adaptation requirements in coastal areas. South, south-east and east Asia are especially prone to these issues, reflecting the dense coastal population and high subsidence potential of deltas and alluvial plains around these coasts^3,8,9^.

The mechanisms of land subsidence are complex, resulting from a multitude of natural processes (e.g. natural compaction) and anthropogenic processes (e.g. underground fluid withdrawal)^5,10^. Anthropogenic causes have the potential to greatly accelerate natural subsidence processes to rates often exceeding 1 cm/yr, with more than 10 cm/yr locally observed in Jakarta, Indonesia. The loss of elevation contributes to RSLR, increasing the risk of coastal erosion, flooding, waterlogging and saltwater intrusion in coastal zones^5,11–13^. Reducing human-induced subsidence (henceforth termed subsidence control) by removing its causes, such as reducing or stopping groundwater withdrawal, has been successfully implemented in several coastal cities, most notably Tokyo^3,6^.

**Fig. 1:**
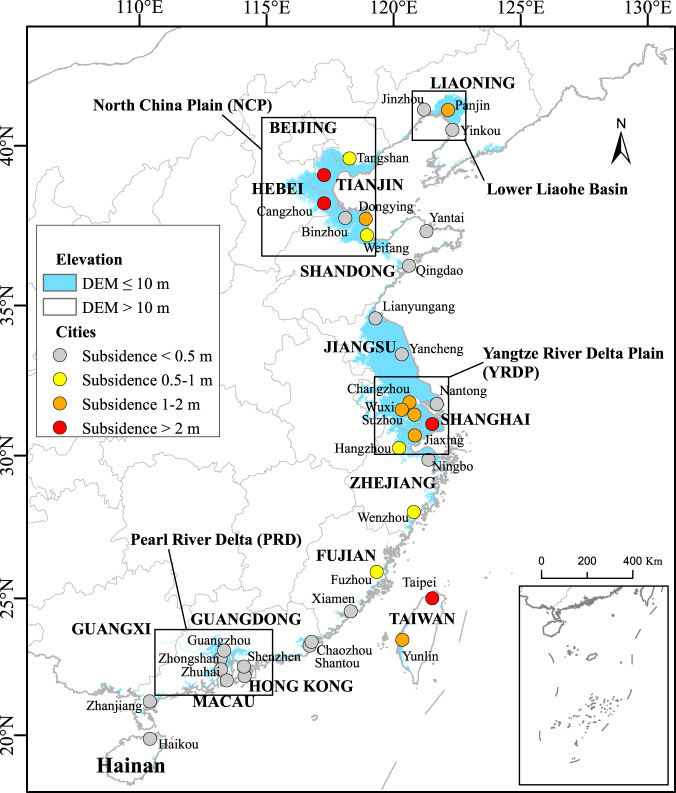
Observed anthropogenic subsidence of 36 coastal cities in China. The figure includes the low-elevation coastal zone (<10-m elevation), the major delta plains, and the observed maximum cumulative anthropogenic subsidence of 36 major coastal cities over the 20th/early 21st century that are considered in the analysis.

China has the largest coastal population in the world^14,15^ and a high subsidence potential along much of its coast due to the presence of many deltas and alluvial plains. These include four major plains (Fig. 1) (a) the Yangtze River Deltaic Plain (YRDP), which includes the Suzhou-Wuxi-Changzhou region, Shanghai and Hangzhou-Jiaxing-Huzhou region; (b) the North China Plain (NCP), including Yellow River Delta (YRD); (c) the Pearl River Delta (PRD) in Guangdong Province; (d) the Lower Liaohe River in Liaoning Province; as well as numerous smaller plains and extensive and growing areas of a land claim, which also often subside. However, the most intense human-induced subsidence happens in cities, relating to rapid urbanisation, economic development and industrialisation and over-exploitation of groundwater. It was first observed in the coastal cities of Shanghai and Tianjin in the early 20th century^16,17^. Subsidence now affects more than eight percent of China’s land area (or 790,000 km^2^), with average cumulative land subsidence of more than 20 cm (in 2009)^18,19^. This includes substantial subsidence in 36 economically-significant coastal cities which are located completely or partly on deltaic/alluvial deposits at elevations below 10 m^17^ (Fig. 1).

Coastal subsidence research in China is strongly focused on Shanghai (e.g.^20–25^,) with less consideration elsewhere or nationally. Here we address this gap and provide a first national-level assessment of the implications of coastal subsidence on RSLR and coastal flooding in China, including the benefits of subsidence control. To do this, we use a bespoke national model of coastal China^26^ developed within the Dynamic and Interactive Vulnerability Assessment (DIVA) model framework comprising more than 2700 segments to represent China’s coast (Supplementary Fig. 1). We add a new national dataset on subsidence in China derived from the literature which comprises contemporary estimates of natural vertical land movement, and observed subsidence in selected deltas and coastal cities (see Supplementary Methods and Supplementary Data 1). Following the methods used in ref. 3, four components of relative sea-level change are considered in addition to climate-induced sea-level change, namely (a) GIA, (b) tectonic movement (comprising subsidence and uplift), (c) deltaic subsidence and (d) additional (human-induced) city subsidence (see Methods and Supplementary Information). (Subsidence in areas of land claim is not considered due to the absence of national datasets). To illustrate the relative effects of these different components on RSLR and its consequences, they are analysed via a series of RSLR assumptions which progressively add components. These should not be confused with RSLR scenarios, as only assumptions 4, 5 and 6 (see Methods), which include all the components are plausible scenarios.

To estimate the national-average RSLR in 2015, we weigh the data by coastal length and by coastal population^3^. These two methods show the average RSLR by coastal length and in terms of coastal residents’ experience of RSLR, respectively. We also assess the relative role of subsidence to future coastal flood risk to 2050 compared with other changes, including climate change (sea-level rise derived from the Representative Concentration Pathways, RCPs), socio-economic change (population and economy derived from the Shared Socioeconomic Pathways, SSPs) and subsidence control and adaptation (via protection). We consider a timeframe to 2050 as it is highly relevant to today’s policies. The future evolution of human-induced subsidence is a complex phenomenon which will depend critically on interactions between a number of drivers, including future human behaviour. It is not presently possible to predict this evolution^2^ and as a first analysis of the scale of the issue, we extrapolate the observed subsidence observations to 2050 as a plausible uncontrolled subsidence scenario^3^. As China is actively engaged with measures to reduce human-induced subsidence in coastal cities, we also explore the benefits of a national “subsidence control” policy in cities (see Supplementary Table 2). All our subsidence scenarios are derived from national guidelines and assume either continuation of recent trends, or full implementation of subsidence control (refs. 19, 25; see Methods for more details). Hence, the analysis provides insights on sensitivity rather than detailed projections.

We also explore two adaptation strategies following current practice in China, which focuses on coastal protection: (a) no further upgrade (maintain dikes at 2015 height) and (b) maintain protection standard (dikes are raised with RSLR).

The following indicators relevant to coastal flood risk until 2050 are analysed: (a) the magnitude of RSLR, (b) floodplain area, (c) floodplain population, (d) value of floodplain assets, (e) expected annual damage to assets, (f) expected annual number of people flooded and (g) protection costs, comprising the sum of capital costs and maintenance costs for dikes (see Methods).

## Results

### Relative sea-level rise

**Fig. 2:**
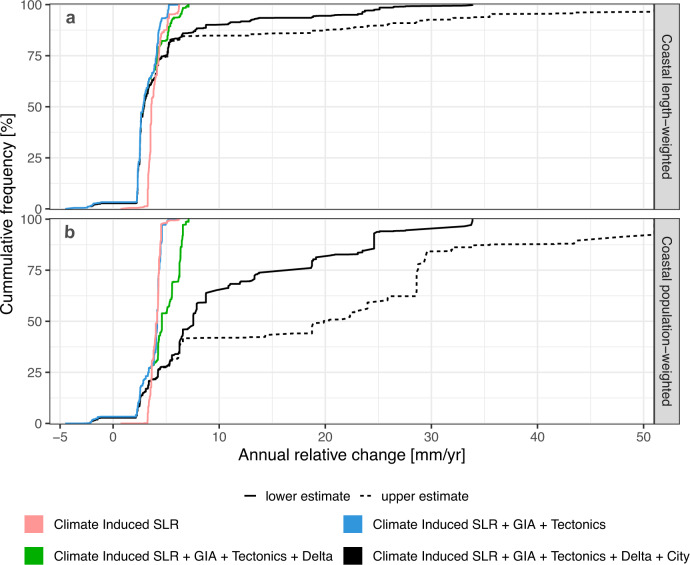
Cumulative distribution of contemporary coastal relative SLR (for the year of 2015). **a** Length-weighted, **b** Population-weighted. Each panel shows climate-induced SLR alone, and then progressively adds the other RSLR components comprising: (1) GIA and tectonics, (2) GIA, tectonics and delta subsidence combined and (3) GIA, tectonics, delta subsidence and uncontrolled city subsidence combined. For uncontrolled city subsidence, uncertainty is considered via a low and high estimate.

Climate-induced SLR in 2015 averaged about 3.9 mm/yr along the Chinese coast (Fig. 2 and Table 1). GIA and tectonic movement by themselves cause a net average fall in RSLR (−0.9 mm/yr). On the national level, delta subsidence in the four major plains increases RSLR along nearly 20% of the Chinese coast and affects about 70% of the coastal population, reflecting the dense population in these areas (Fig. 2). Adding city subsidence significantly increases RSLR. Unlike the global case^3^, in which the length-weighted city subsidence is barely visible, about 40% of the Chinese coast is urbanised and subsiding, including smaller coastal plains and deltas. Nearly 90% of the coastal population is affected by this subsidence. In total, considering all these components, the average RSLR for China in 2015 is 4.9 to 7.7 mm/yr (length-weighted) and 10.9 to 20.0 mm/yr (population-weighted). Hence, every Chinese coastal resident experiences an average RSLR that is two to three times higher than length-weighted RSLR and three to five times higher RSLR due to climate-induced SLR alone (Table 1). This reflects that coastal residents are concentrated in subsiding areas and the effect of subsidence on RSLR is much higher in China than globally^3^. Just considering climate-induced sea–level rise in China significantly underestimates the contemporary magnitude of RSLR and especially human impacts.

**Table 1:** The contemporary contribution of the climate and geologic components to relative sea-level rise for length-weighted and population-weighted cases, respectively (for the year 2015)

Relative SLR component	Contribution to relative sea-level change			
	Coastal-length weighted		Coastal-population weighted	
	mm/yr	%	mm/yr	%
Climate-induced SLR	3.9	81 to 51	4	20 to 37
GIA + Tectonics	−0.9	−18 to −11	−0.3	−2 to −3
Delta subsidence	0.2	4 to 3	0.9	4 to 8
City subsidence	1.7 to 4.5	35 to 58	6.3 to 15.4	58 to 77
National-mean sum	4.9 to 7.7		10.9 to 20	

From 2015 to 2050, we also analysed RSLR assumptions considering three RCP-derived climate-induced SLR scenarios and assuming the other four components remaining constant using length-weighted averaging (Supplementary Fig. 4). Only considering climatic factors, the range of SLR in China is 11–27 cm in 2050 (reference year is 2015, Supplementary Table 3), with the mean SLR rate at 5.1 mm/yr between 2015 and 2050. Considering all the RSLR components, including uncontrolled high city subsidence, RSLR is significantly higher (Supplementary Fig. 4), with a range from 17 to 40 cm in 2050, with a mean RSLR rate of 8.8 mm/yr between 2015 and 2050. This is 73% higher than climate-induced SLR alone. If city subsidence is controlled, the RSLR range in 2050 is 10–26 cm with a rate of 4.8 mm/yr, which is similar to the climate-only assumption due to the offsetting effects of GIA and tectonics, which are causing uplift. Under the highest RSLR assumption (i.e. with maximum uncontrolled city subsidence), the contribution of climatic factors to RSLR is 57% and that of non-climatic factors is 43% at the national level in 2015. With subsidence control, this distribution changes and the contribution of climatic factors to RSLR is 93% and non-climatic factors are reduced to 7% in 2015. This demonstrates that subsidence control measures can be effective, in theory,  if implemented quickly.

### Flood exposure

**Fig. 3:**
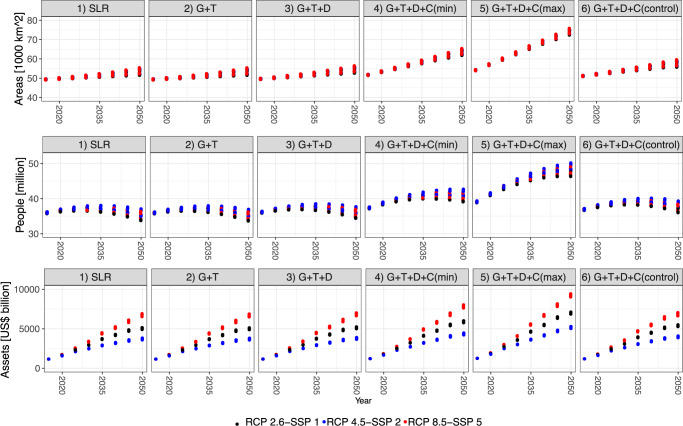
Potential floodplain extent, population and assets to a 100-year extreme coastal flood event under RCP-SSP scenarios between 2015 and 2050. RSLR assumptions as indicated: (1) climate-induced SLR only; (2) climate-induced SLR, GIA and tectonics; (3) climate-induced SLR, GIA, tectonics and delta subsidence; (4) climate-induced SLR, GIA, tectonics, delta and minimum uncontrolled city subsidence; (5) climate-induced SLR, GIA, tectonics, delta and maximum uncontrolled city subsidence; and (6) climate-induced SLR, GIA, tectonics, delta and controlled city subsidence.

We calculate flood exposure in terms of land area, people and assets exposed to an indicative extreme event (the 100-year flood), excluding the effects of coastal protection for all six RSLR assumptions. This allows the effect of the different RSLR components to be seen. Land areas and exposed assets will increase significantly by 2050 in all cases. The exposed population increases to the 2030s and then slightly decreases by 2050, except for the highest assumption when it continues to increase (Fig. 3). The decreasing population trend despite SLR reflects demography—there is a decline of China’s total population under all SSPs^26^.

By 2050, under climate-induced SLR only (Assumption 1), 53,190 km^2^ of land, 35 million people and US$ 5,263 billion of assets are exposed to the 100-year coastal flood event (Supplementary Table 4). With all RSLR components, including uncontrolled city subsidence (Assumptions 4 and 5), the 100-year coastal floodplain will expand to 64,000–74,000 km^2^ in 2050 (an increase of 20–39%). The largest population in the 100-year coastal flood event is under RCP 8.5-SSP 5 (50 million), with associated assets of US$ 9,566 billion. Population and assets exposed to the 100-year coastal flood event under the highest subsidence assumption is about 36–39% higher than in the climate-induced SLR-only assumption. Subsidence control (Assumption 6) is an effective response to reduce growth in exposure to coastal flooding: the increase compared to the climate-induced SLR only assumption is only 7% for exposed land, 6% for exposed population and 7% for exposed assets, respectively.

### Coastal flood risk

Flood risk also considers the influence of flood protection which is estimated with the DIVA model^26,27^. Assuming no upgrade of protection from 2015 to 2050, the expected number of people flooded annually is highest under RCP 8.5-SSP 5 and lowest in RCP 2.6-SSP 1. The annual flooded population will be higher in 2050 than in 2030 despite the declining population exposure (Supplementary Fig. 5). Flood damage costs under RCP 8.5-SSP 5 are three times higher than RCP 2.6-SSP 1 in 2050. The average annual flood cost will be about US$ 102 billion per year in 2030. This increases by ~10–23 times to US$ 1076–2374 billion per year by 2050 under the highest RSLR assumption. In contrast, if current protection standards are maintained with RSLR, these damages are reduced to US$ 33–61 billion flood damage per year at a extra protection cost of US $2 to $4 billion per year, depending on the scenario. Applying subsidence control further reduces damage to US$ 25-46 billion per year.

Maintaining current protection standards costs US$ 8–10 billion per year under RCP 2.6-SSP 1 in 2050, depending on the magnitude of human-induced subsidence (Table 2). Considering all RSLR components lead to higher protection costs than due to climate-induced SLR alone, but the increase in protection cost is small (up to US$ 2 billion/year) compared to the reduction in flood damage costs. Across all the RSLR assumptions, the increased dike costs are at least two orders of magnitude lower than the avoided flood damage costs, even without considering indirect damages.

**Table 2:** Flood damage costs and adaptation (dike) costs in 2050 based on the RCP 2.6-SSP 1 scenario (median values)

	(US$ billion/yr)					
RSLR assumptions	Flood damage costs		Dike costs		Difference between the adaptation strategies	
	No upgrade	Maintain protection standard	No upgrade	Maintain protection standard	Reduced flood damage costs	Increased dike costs
1) Climate-induced SLR only	81	32	6	8	49	2
2) Climate-induced SLR + GIA + Tectonics	75	31	6	8	44	2
3) Climate-induced SLR + GIA + Tectonics+Delta	99	32	6	8	67	2
4) Climate-induced SLR + GIA + Tectonics+Delta+City (min)	717	38	6	9	679	3
5) Climate-induced SLR + GIA + Tectonics+Delta+City (max)	1553	46	6	10	1507	4
6) Climate-induced SLR + GIA + Tectonics+Delta+City (control)	336	34	6	8	302	2

With subsidence control, people who are flooded and damage costs are significantly reduced compared to uncontrolled subsidence. For no upgrade, damage costs are reduced to 22 to 47%, while for upgraded protection costs are reduced to 74 to 89% (Table 2).

**Fig. 4:**
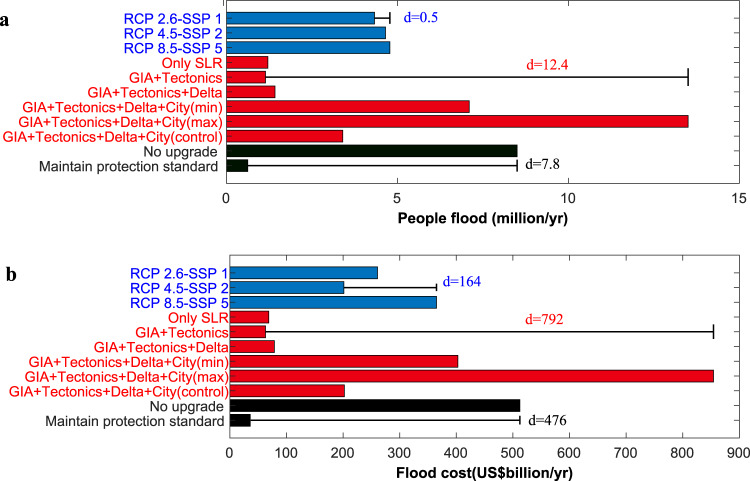
Sensitivity of impacts in 2050 to the uncertainty dimensions considered. **a** People flooded; **b** Flood cost. Sensitivity is calculated as the average difference (d and error bars) between the impacts by modifying one input variable while holding the other dimensions constant.

To explore how much flood impacts are affected by changes in protection strategies and RSLR assumptions, a sensitivity analysis (one-driver-at-a-time approach) is conducted^28,29^. This shows that impacts are most sensitive to the RSLR assumption, which is much larger than the changes caused by RCP-SSPs in 2050 (Fig. 4). As subsidence control is being implemented in many places in China, if we exclude the pessimistic RSLR assumption (i.e. maximum city subsidence assumption), then flood impacts are most sensitive to the adaptation strategy in terms of people flooded and flood damage cost. The differences caused by the adaptation strategy and by the subsidence contribution are in the same order of magnitude.

These results indicate that the effect of coastal protection (i.e. adaptation) is as significant as subsidence control. To effectively manage the impacts caused by climate-induced SLR and subsidence, China needs to continue upgrading coastal protection measures and implement subsidence control under all the RCP-SSP scenario combinations.

## Discussion

Our results emphasise that subsidence is a significant threat to China’s coastal environment and human well-being. Rather than just being a local problem, anthropogenic subsidence is nationally significant as people preferentially live in the subsiding areas (Fig. 2)^3^. Compared with natural subsidence, which accumulates over centuries and longer, anthropogenic subsidence appears more local and is often much more rapid. Hence, the effects of anthropogenic subsidence are visible over relatively shorter timescales (i.e. decades). It reduces the effective protection levels of dikes, increases maintenance costs and amplifies the consequences of failure of flood protection infrastructure. For example, subsidence in Shanghai city has required the flood defence walls along the Huangpu River to be raised four times since 1959, with a total increase in the height of 3.4 m in the Bund area, requiring large expenditure and enhancing residual risk^30^. Urban areas and population in coastal floodplains increased rapidly in China from 1990 to 2015^31,32^, and this is projected to continue to increase until 2030^26^. Thus human-induced subsidence has a strong potential to continue over the next few decades as explored here and maybe longer: even with falling populations, coastal economic activity is still likely to increase, potentially maintaining and even enhancing water demand^33^.

The impacts assessed in this study only consider the flood impacts of land subsidence, ignoring other damages that might occur, such as building collapse, and the formation of local subsidence bowls, ground cracks or fissures^17,34^. Subsidence can also lead to saline intrusion and waterlogging, impacting water quality, ecosystem services and agriculture^35,36^.

While not considered in this analysis due to a lack of systematic data, land subsidence and deformation is also observed widely in new coastal reclamations, such as in Macau, Hong Kong, Shenzhen, Shanghai, Tianjin and Xiamen (e.g. refs. 24,37–39). With considerable further land reclamation planned in coastal China^40^, these issues may continue and even grow in the coming decades, without consideration of these issues in the design of new reclamations^41^.

In conclusion, this research shows it is essential to understand and address subsidence and the resulting RSLR across coastal China. Traditionally, subsidence is considered to be a local problem. This study demonstrates subsidence has national implications and there is a need for a national policy response: subsidence control and higher dikes reflecting current policy approaches are considered here, and a wider range of adaptation measures across protection, accommodation, retreat and advance^1^ could be considered in more detailed analysis. More detailed national and regional assessments of flooding and subsidence are recommended, including the costs and benefits of management in the context of climate-induced sea-level rise. Nicholls et al.^3^ have shown that the issues raised in this work have global significance, particularly in south, south-east and east Asia. Similar assessments across these Asian nations, more systematic collection of subsidence data, and more efforts to better predict future subsidence would facilitate an appropriate response to this issue.

## Methods

### Data, assumptions and scenarios

The analysis has two distinct parts: (a) a national analysis of contemporary RSLR based on integrating estimates of climate-induced and geological components; and (b) an analysis of coastal flood risk to 2050, including climate and socio-economic change, uncontrolled versus controlled subsidence and protection scenarios. This makes various assumptions about RSLR, including that contemporary rates continue to 2050 versus subsidence control is fully implemented.

We use the DIVA coastal flood module^28,42^ for the analysis. DIVA is an integrated, state-of-the-art model of coastal systems that assesses biophysical and socio-economic consequences of sea-level rise and socio-economic development, which has been applied to problems such as coastal erosion^43^, coastal flooding^28^, coastal wetland change^44^, subsidence and relative sea-level rise^3^ and coastal migration^45^ among others. The underlying structure is a global dataset of coastal areas and floodplains based on 12,148 coastal segments which divide the world’s coast (excluding Antarctica). However, for this application, a higher resolution, more detailed, national database for the Chinese coast is used. It comprises 2760 coastal segments, covering 28,966 km of coastline^26^. Within DIVA, coastal protection is modelled by means of dikes. Protection cost is based on standard unit costs by length and height raised^28^ with annual maintenance costs of one percent of the (accumulated) capital investment.

The SLR observations are from the European Space Agency Climate Change Initiative Sea Level Project:^46^ here, we use SLR observations from 2010 to 2015. Future SLR scenarios from 2015 to 2050 follow RCP 2.6, RCP 4.5 and RCP 8.5 emissions and are from ref. 28. They are consistent over this timeframe to the SLR scenarios of the Intergovernmental Panel on Climate Change, Sixth Assessment Report^47^. The SSPs represent a range of plausible socio-economic changes including population^48^ and GDP^49^. The RCP-SSP combinations considered are applied to all the RSLR assumptions, so this aspect of the analysis is a guided sensitivity analysis rather than a scenario analysis. The AW3D30 DEM dataset^50^ and LandScan population dataset^51^ have been used to build the DIVA-China database.

Three main factors leading to natural subsidence and uplift are considered: GIA, tectonic movement, and natural compaction. The GIA and tectonic movement may cause loss or gain in land elevation^5,52^. In China, most of the coastal rocky mountainous regions are uplifting at annual uplift rates of ~0-5 mm/yr, and subsidence due to neotectonic movement is about 1–3 mm/yr (Supplementary Fig. 2)^53^. Natural compaction in deltas is typically less than 3 mm/yr^54^. Subsidence due to tectonism and natural settlement of under-consolidated soils occurs widely in coastal China, but at a slow, relatively uniform rate, which is unlikely to exceed 5 mm/yr, except an abrupt situation such as during strong earthquakes (which are not considered here). This is less than subsidence caused by human factors. Further, natural subsidence cannot be controlled or mitigated, and protection (i.e. adaptation) is the only response to the impacts. Note that controlled flooding of delta plains to enhance sedimentation can be a response^3^, although this is not considered here.

In coastal China, the major contribution to land subsidence is anthropogenic factors, such as (a) excessive withdrawal of underground fluids, including groundwater, oil and gas; (b) extraction of coal and ores; (c) underground excavation for tunnels and caverns and (d) compaction due to drainage and the load of buildings^5,52,54^.

To analyse the magnitude and impacts of subsidence on SLR in China, we combine data on four components of relative sea-level change, (a) GIA, from ref. 55; (b) Tectonic subsidence/uplift, derived and verified from multiple sources (Supplementary Fig. 2); (c) Delta subsidence, with natural compaction being considered in four deltas (Supplementary Table 1); (d) City subsidence, which represents the additional subsidence beyond GIA, tectonic and delta subsidence that coastal cities in deltaic and alluvial plains experience due to human causes^3^. Given the wide range of values of subsidence reported, a low and a high estimate of the mean additional city subsidence rate is developed to represent the uncertainty (Supplementary Table 2). We synthesise the available subsidence rates of deltas and coastal cities and assign subsidence rates (mm/yr) to the appropriate coastline segments in the DIVA model^3,28,42^. This means that the subsidence estimates are not applied to an entire city unless this is appropriate (Supplementary Fig. 3). To estimate the effects of subsidence control, we assume controlled subsidence rates consistent with the goals set by the Chinese government in the “National Land Subsidence Prevention and Control Programme (NLSPCP) (2011-2020)^19^” (Supplementary Table 2). These are consistent with global estimates of what is possible to achieve with subsidence control^3^. Thus, six RSLR assumptions can be considered:

Assumption 1: Climate-induced SLR only: only consider climatic drivers, without any subsidence and uplift;

Assumption 2: Climate-induced SLR + GIA + Tectonics: only consider natural processes, including GIA and tectonic movement (Supplementary Fig. 3a);

Assumption 3: Climate-induced SLR + GIA + Tectonics+Delta: including GIA, tectonic movement and deltaic subsidence;

Assumption 4: Climate-induced SLR + GIA + Tectonics+Delta+City (min): including GIA, tectonic movement, deltaic subsidence and minimum estimate of additional city subsidence (Supplementary Fig. 3b and Supplementary Table 3). City subsidence continues at current rates from around 2010 to 2050;

Assumption 5: Climate-induced SLR + GIA + Tectonics+Delta+City (max): including GIA, tectonic movement, deltaic subsidence and maximum estimate of additional city subsidence (Supplementary Fig. 3c and Supplementary Table 3). City subsidence continues at current rates from around 2010 to 2050. This is the highest RSLR assumption.

Assumption 6: Climate-induced SLR + GIA + Tectonics+Delta+City (control): including GIA, tectonic movement, deltaic subsidence and additional city subsidence, assuming control measures are implemented to reduce subsidence rates. These follow the city subsidence rates defined in Supplementary Table 3 from 2010 to 2050.

### Flood risk assessments

The 100-year extreme water level estimates come from ref. 56 beyond 2015, these water levels are raised with the RSLR scenario. We follow earlier DIVA analyses and current practices in China by modelling coastal protection (i.e. adaptation) by means of dikes^26–28^. Dikes are initialised in 2010 with construction costs of US$ 644 billion^26^. There are two protection strategies for 2050: (a) maintain the protection standard and (b) no upgrade of protection (maintaining current dike heights). To maintain the protection standard, dikes are maintained at the current protection level over time (i.e. they are raised by RSLR), requiring capital investment. For no upgrade, the dikes are maintained at 2015 dike heights and become increasingly less effective protection against coastal flooding as sea levels rises. In addition, protection infrastructure requires annual maintenance (estimated at one percent of capital cost)^26^. Three RCP-SSP combinations (RCP 2.6-SSP 1, RCP 4.5-SSP 2 and RCP 8.5-SSP 5) were selected to define climate-induced sea-level rise, demographic and economic scenarios and assess RSLR, exposed land, population and assets to coastal flood, as well as flood damages and adaptation costs^57^. The costs of subsidence control are not considered. Flood damages are calculated using depth-damage relationships with assets estimated using GDP/density. A more detailed description of the coastal flooding module is given in ref. 26 and ref. 28.

Sensitivity is calculated as the average difference between the impacts in 2050 by modifying one input variable, while holding the other variables constant, following ref. 28 and ref. 29.

## Supplementary information

Supplementary Information

Description of Additional Supplementary Files

Supplementary Data 1

## Data Availability

All datasets used in the production of this paper are available from: 10.5281/zenodo.6969115^58^. Source data are provided with this paper. Creative Commons Attribution 4.0 International Public License.

## Code availability

The DIVA code is available at https://gitlab.com/daniel.lincke.globalclimateforum.org/diva_published. The R code used to produce the numbers, tables and figures is available from: 10.5281/zenodo.6969115^58^. Source data are provided with this paper. Creative Commons Attribution 4.0 International Public License.
