# Supplementary material for: Benefits of subsidence control for coastal flooding in China
Source: Nat Commun. 2022 Nov 14;13:6946. doi: 10.1038/s41467-022-34525-w (PMC9663704; doi:10.1038/s41467-022-34525-w)
Supplement: Supplementary file 1 — Supplementary Information [file 41467_2022_34525_MOESM1_ESM.pdf]

Supplementary Information for

Benefits of subsidence control for coastal flooding in China

Jiayi Fang[1,2,3,4,5]*, Robert J. Nicholls[5,6]*, Sally Brown[5,6], Daniel Lincke[7], Jochen Hinkel[7,8], Athanasios T. Vafeidis[9], Shiqiang Du[10], Qing Zhao[4], Min Liu[4], Peijun Shi[3,11]*

1. Institute of Remote Sensing and Earth Sciences, Hangzhou Normal University, Hangzhou, 311121, China
2. Zhejiang Provincial Key Laboratory of Urban Wetlands and Regional Change, Hangzhou, 311121, China
3. Academy of Disaster Reduction and Emergency Management, Ministry of Emergency Management & Ministry of Education, Beijing 100875, China
4. School of Geographic Sciences, East China Normal University, Shanghai, 200241, China
5. School of Engineering, University of Southampton, Southampton, SO16 7QF, UK
6. Tyndall Centre for Climate Change Research, University of East Anglia, Norwich Research Park, Norwich, NR4 7TJ, UK
7. Global Climate Forum e.V. (GCF), Berlin, 10178, Germany
8. Division of Resource Economics, Albrecht Daniel Thaer-Institute and Berlin Workshop in Institutional Analysis of Social-Ecological Systems (WINS), Humboldt-University, Berlin, 10099, Germany
9. Coastal Risks and Sea-Level Rise Research Group, Department of Geography, Christian-Albrechts-University Kiel, Kiel, 24098, Germany
10. School of Environmental and Geographical Sciences, Shanghai Normal University, Shanghai, 200234, China
11. State Key Laboratory of Earth Surface Processes and Resource Ecology (ESPRE), Beijing Normal University, Beijing, 100875, China

*Authors for corresponding: Robert J. Nicholls <**Robert.Nicholls@uea.ac.uk**>, Jiayi Fang <jyfang822@foxmail.com>, Peijun Shi <spj@bnu.edu.cn>

This PDF file includes:

Supplementary Methods
Supplementary Fig. 1-5
Supplementary Table 1-4
Legends for Supplementary Data 1
Supplementary References

Other supplementary materials for this manuscript include the following:

Supplementary Data 1

Supplementary Methods

Supplementary Fig. 1 provides a flowchart summarising the general methodological approach as explained in the methods summary. It comprises three main steps. The first step is to develop the spatial assessment units by using a detailed coastline data and a segmentation process. The produces a detailed segmentation of China's coast, comprises 2,760 coastal segments, covering 28,966 km of coastline. This is much more detailed than the global database used by Hinkel et al (1) and Nicholls et al (2) as explained by Fang et al (3). The second step is to populate the segment database with baseline exposure parameters (in 2015) using elevation and population datasets. This creates a database structure that enables the DIVA model to run. These first two steps were completed by Fang et al (3), and the same segmentation and data is used in this analysis. In the third step, the DIVA coastal flooding module is used to assess present and future changes of relative sea-level rise and how coastal flood risk evolves for different assumptions and scenarios. This includes considering the implementation of subsidence control in the 36 coastal cities and/or adaptation strategies via protection with dikes. The scenarios of climate-induced sea-level rise and population change and economic growth are consistent with the RCPs and SSPs as explained in the main text. The adaptation scenarios consider protection in terms of no upgrade, or upgraded protection to maintain the current standard with relative sea-level rise. This analysis adds the new data on the uplift/subsidence scenarios and hence the RSLR assumptions, as well as considering subsidence control scenarios based on official government documents. This makes the analysis much more realistic of what is happening in coastal China today.

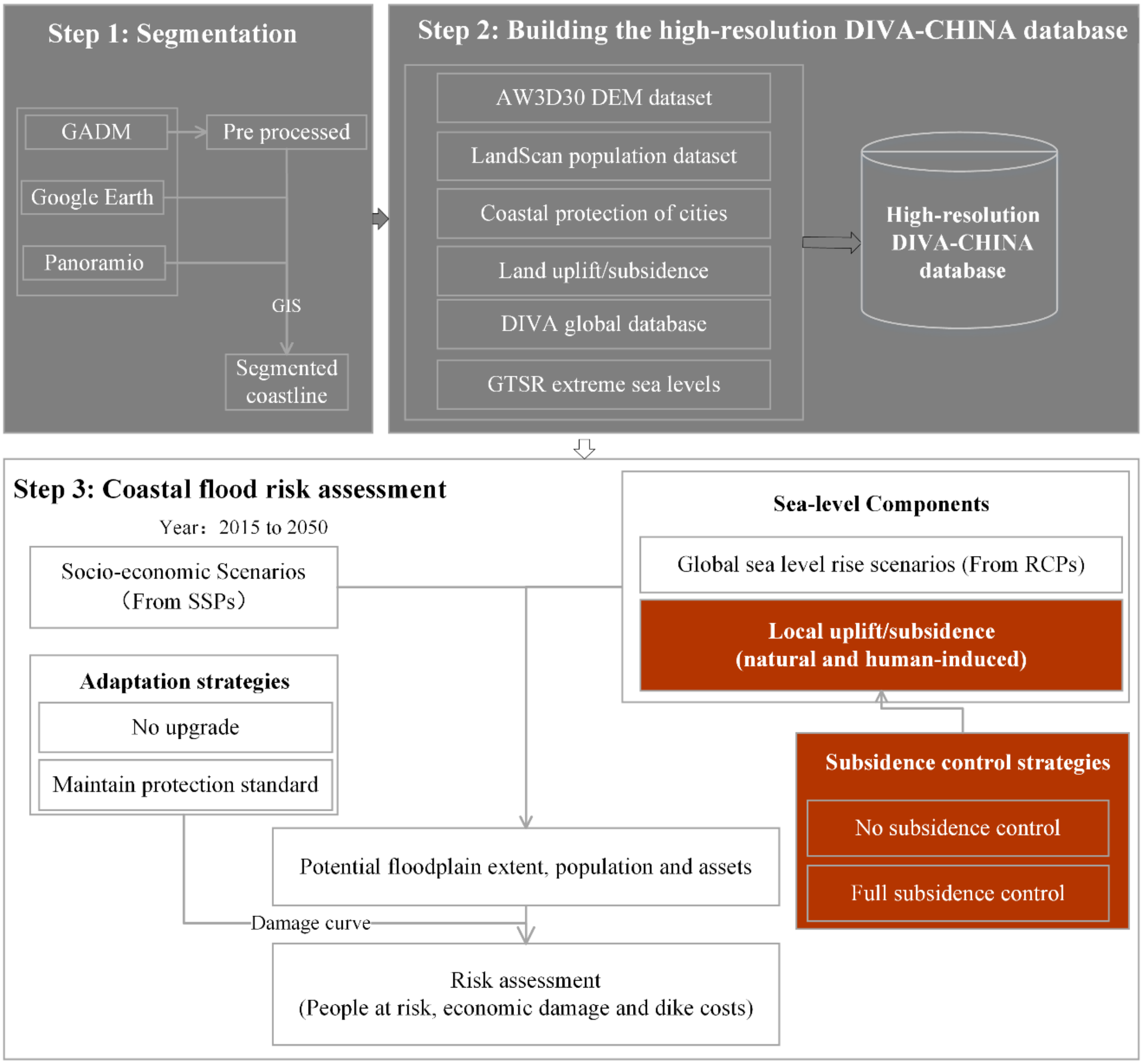

Supplementary Fig. 1: Flowchart summarising the general methodological approach used in this paper. Steps 1 and 2 were completed by Fang et al (3), while Step 3 is augmented by new information on relative sea-level rise due to a range of sources, and includes strategies for subsidence control in subsiding coastal cities. These additions are coloured in red.

To analyse the effects of subsidence on relative sea-level rise in China, we combine data on four components of relative sea-level change, which are described in more detail in subsections below:

1. Glacial-isostatic adjustment (GIA);
2. Tectonics subsidence/uplift, derived and verified from multiple references (Supplementary Fig. 2);
3. Delta subsidence, reflecting natural compaction in four large deltas (Supplementary

Table 1);

4. City subsidence, which captures the additional subsidence beyond delta subsidence that coastal cities located on deltaic and alluvial plains experience. We consider 36 coastal cities that are prone to subsidence with populations over 1 million (except Macau and Yunlin, Taiwan) (Supplementary Table 2).

These four components are independent and hence can simply be summed for each segment.

Glacial Isostatic Adjustment (GIA)

Local sea-level change due to glacial isostatic adjustment caused by ice loading and unloading are taken from the ICE-6G_C (VM5a) model (4). This gridded dataset is projected to the DIVA coastal segments by assigning the average sea-level change value over all intersected grid cells to the relevant segment.

Tectonic subsidence/uplift

Tectonic subsidence/uplift was derived and verified from multiple references, mainly from Li and Feng (5), Lu and Ding (6), Feng et al. (7) and Cai et al. (8). The eastern coast of China is located on the western margin of the western Pacific tecto13nic belt, and neotectonic movement is significant since the Quaternary (5, 6). Sixteen distinct geological regions were distinguished as defined in Supplementary Fig. 2. Half these regions are experiencing uplift and half are subsiding. Most of the coastal rocky mountainous regions are slowly uplifting at ~0-5 mm/yr, while subsidence due to neotectonic movement in the lower areas is about 1-3 mm/yr (7, 8). The values used in this analysis are shown in Supplementary Fig. 2.

Delta subsidence

Natural compaction of deltaic sediments involves natural changes in the void space within sedimentary layers (for example dewatering, grain-packing realignment and organic matter oxidation) and is typically ≤3 mm/yr (9). For the four main delta plains in China, delta subsidence varies from 1-5 mm/yr (10-12). For the Liaohe River Delta, where there is little or no data for natural delta subsidence, a minimum value of

subsidence is assumed in all cases at 1 mm/yr, following the estimates of Mechel et al. (13). The delta extent is linked to the DIVA segments. Supplementary Table 1 summarises the deltas considered in the analysis and the subsidence values used.

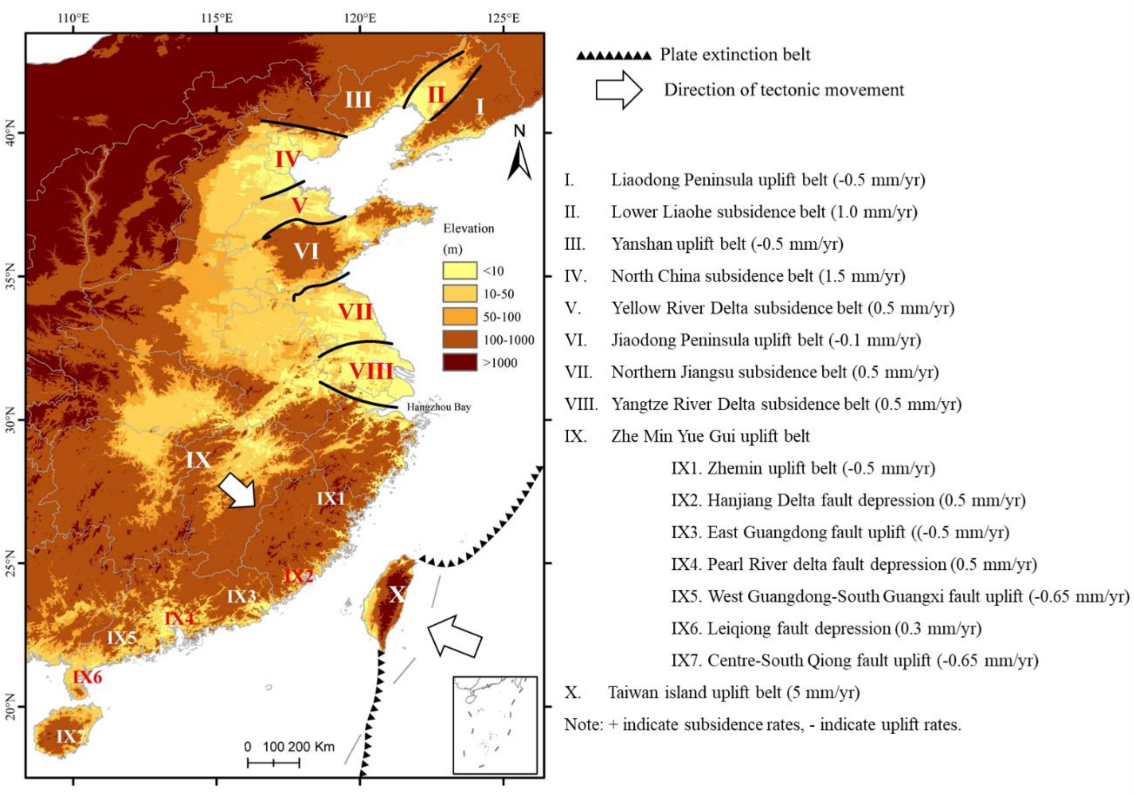

Supplementary Fig. 2: The tectonic movement of coastal China, indicating the 16 different basins and units identified in this analysis. Positive values indicate subsidence and negative values indicate uplift. Data sources: Li and Feng (5), Lu and Ding (6), Feng et al. (7) and Cai et al. (8).

Supplementary Table 1: Published subsidence rates due to observed delta-wide compaction in the major delta plains in China and the values applied in this study: a positive value indicates subsidence.

Delta Plain	Subsidence (mm/yr)	Source	Deltaic subsidence used in this study (mm/yr)
Yellow River	1.8	Ericson et al. (14)	3.0
	5	Shi et al. (15);<br>Tan et al. (16);<br>Zhang et al. (17);	
	3-4	Ren (10)	
	2.7	Zhang et al. (18)	
Yangtze River	2.1	Ericson et al. (14)	2.1
Pearl River	1.5	Ren (10)	1.5
Liaohe River	1.0	Expert Judgement	1.0

City subsidence

Large coastal cities are considered in the analysis as they contain the largest coastal populations and significant subsidence is observed in many of them as summarised in Supplementary Table 2. It is found that of these 36 subsiding cities (Supplementary Data 1), most are situated wholly or partly on deltaic/alluvial deposits which may subside due to the human-induced processes such as subsurface fluid withdrawal and/or drainage. In each case, the additional human-induced subsidence beyond that captured in the delta subsidence estimates (Supplementary Table 1) is estimated based on a survey of the published literature and/or expert judgement if required. Given the wide range of values of subsidence reported for cities, a low and a high estimate of the average subsidence is made to represent the uncertainty following Nicholls et al (2). Sources often report the maximum values of subsidence. However, it is not appropriate to use a maximum localised subsidence value for the whole subsiding area. Instead we develop and use indicative average estimates of subsidence across the subsiding area in each city and then link this to the appropriate DIVA segments (i.e. subsidence is not applied to the entire city unless this is appropriate, Supplementary Fig. 3). These estimates are designed to represent average subsidence values across the whole subsiding area within each city. Supplementary Table 2 summarises the 36 coastal cites that are considered in the analysis and the subsidence values that are used based on the literature. Due to the tremendous damage and public attention caused by human-induced subsidence, a formal assessment called the "National Land Subsidence Prevention and Control Program (NLSPCP) (2011~ 2020)" was conducted by the Chinese government to understand and control subsidence impacts (19). NLSPCP has proposed subsidence control targets for these city subsidence hotspots (Supplementary Table 3). We take these targets as plausible scenarios of subsidence control.

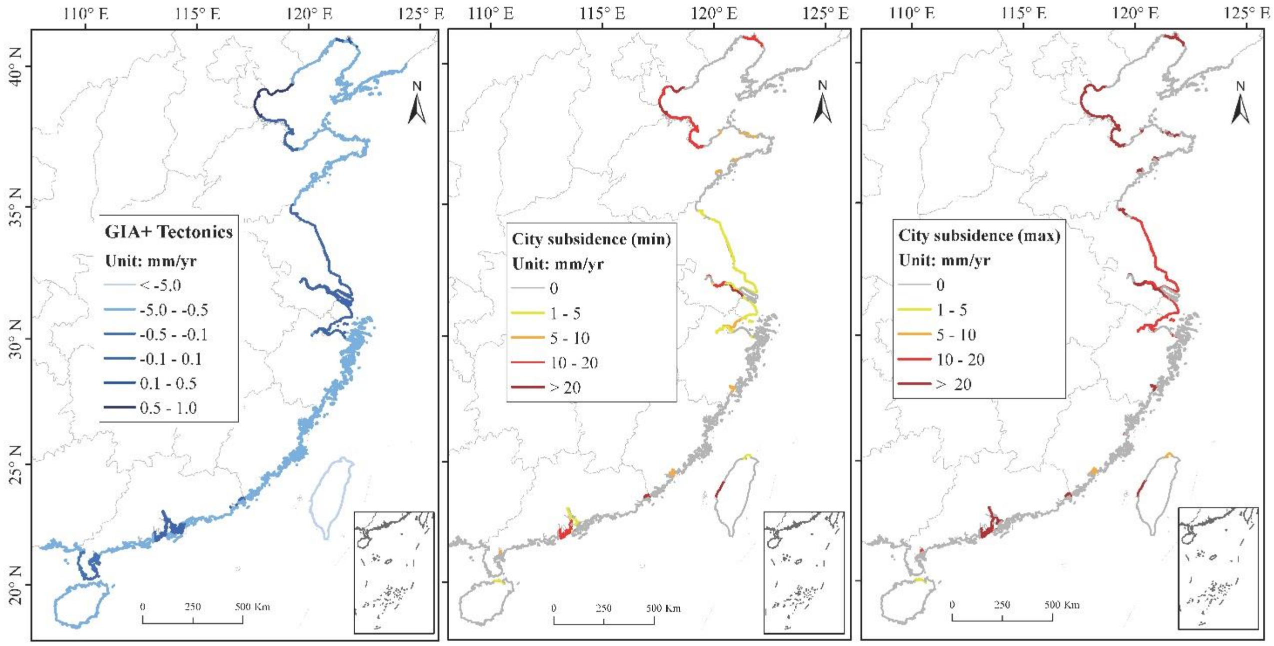

Supplementary Fig. 3: Subsidence/uplift components of China's coastline as input in DIVA. a) GIA and tectonic movement; b) additional minimum city subsidence; c) additional maximum city subsidence.

Supplementary Table 2: Additional uncontrolled and controlled subsidence rates applied by city taken from MLR (19): a positive value indicates subsidence. If cities are not included, no additional subsidence is applied.

Province	City	Population in 2015 (millions)	Subsidence rate (mm/yr)			
			Uncontrolled		Controlled	
			Low	High	2010-2020	2020-2050
Liaoning	Panjin	1.29	18	58	10	5
	Jinzhou	3.02	18	48	10	5
	Yingkou	2.32	18	48	10	5
Tianjin	Tianjin	15.47	20	24	15	5
Hebei	Tangshan	18.25	23	37	20	5
	Cangzhou	7.74	28	43	20	5
Shandong	Dongying	1.91	18	46	10	5
	Binzhou	3.89	12	46	10	5
	Weifang	8.93	12	25	10	5
	Qingdao	7.83	10	70	10	5
	Yantai	6.53	10	50	10	5
Jiangsu	Lianyungang	5.31	4.5	29.5	4.5	4.5
	Yancheng	8.28	4.5	14.5	4.5	4.5
	Nantong	7.67	4.5	19.5	4.5	4.5
	Changzhou	4.70	3.4	37.3	3.4	3.4
	Suzhou	6.67	27.4	47.4	12	5
	Wuxi	4.80	12.4	22.4	12	5
Shanghai	Shanghai	24.15	1.3	17.4	1.3	1.3
Zhejiang	Hangzhou	7.24	2.7	16	2.7	2.7
	Ningbo	5.87	4.1	12	4.1	4.1
	Jiaxing	3.49	9.0	18	9.0	5
	Wenzhou	8.11	9.5	29.5	9.5	5
Fujian	Fuzhou	6.78	5.5	14.5	5.5	5
	Xiamen	2.11	5.5	10.5	5.5	5
Guangdong	Shantou	5.51	23	40	10	5
	Chaozhou	2.73	23	40	10	5
	Shenzhen	3.69	2	23	2	2
	Zhuhai	1.12	18	30	10	5
	Zhongshan	1.59	18	23	10	5
	Zhanjiang	8.23	9.5	18.5	9.5	5
	Guangzhou	8.54	2	24	2	2
Hainan	Haikou	1.65	1	2	1	1
	Hong Kong	7.23	1	10	1	1
	Macau	0.56	3	16	3	3

Special Administrative Region	Taipei, Taiwan	2.70	5	10	5	5
	Yunlin, Taiwan	0.65	23	75	10	5

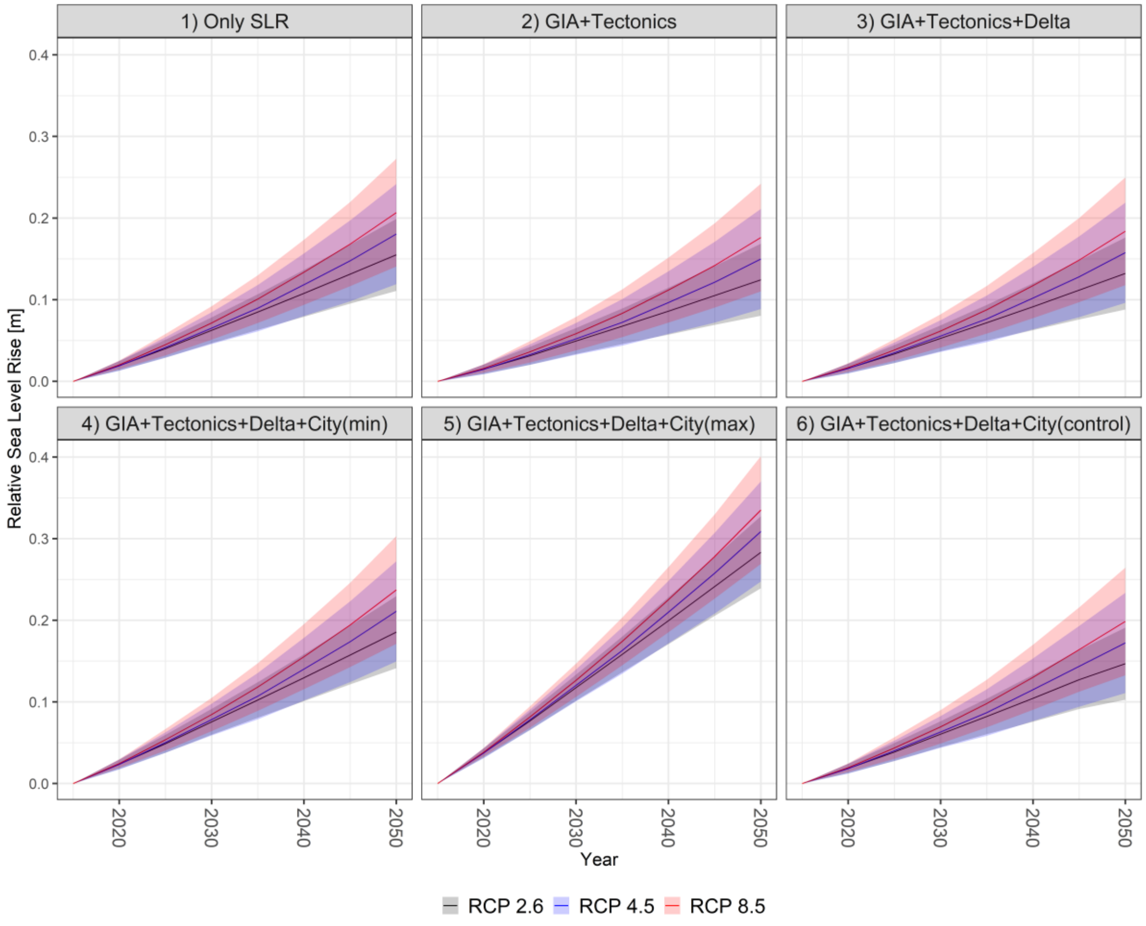

Supplementary Fig. 4: Mean relative sea-level rise in China under RCP2.6, RCP4.5 and RCP8.5 scenarios between 2015 to 2050 by length-weighted method. The median in solid line, the maximum and minimum values in shadow are provided. Subsidence assumptions as indicated: 1) climate-induced SLR only; 2) climate-induced SLR, GIA and tectonics; 3) climate-induced SLR, GIA, tectonics and delta subsidence; 4) climate-induced SLR, GIA, tectonics, delta and minimum uncontrolled city subsidence; (5) climate-induced SLR, GIA, tectonics, delta and maximum uncontrolled city subsidence; and (6) climate-induced SLR, GIA, tectonics, delta and controlled city subsidence (reference period: 2015).

Supplementary Table 3: Relative sea-level rise (meters) in China of 2050 by the length-weighted method. The median and, in parentheses, the maximum and minimum values are provided. Reference year is 2015.

RSLR Assumptions	RCP 2.6	RCP 4.5	RCP 8.5
1) Climate-induced SLR only	0.15 (0.11, 0.20)	0.18 (0.12, 0.24)	0.20 (0.14, 0.27)
2) SLR+GIA+Tectonics	0.12 (0.08, 0.17)	0.15 (0.09, 0.21)	0.17 (0.11, 0.24)
3) SLR+GIA+Tectonics+Delta	0.13 (0.08, 0.18)	0.16 (0.10, 0.22)	0.18 (0.12, 0.25)
4) SLR+GIA+Tectonics+Delta+City (min)	0.21 (0.14, 0.23)	0.21 (0.15, 0.27)	0.23 (0.17, 0.30)
5) SLR+GIA+Tectonics+Delta+City (max)	0.30 (0.23, 0.33)	0.31 (0.25, 0.37)	0.33 (0.27, 0.40)
6) SLR+GIA+Tectonics+Delta+City (control)	0.14 (0.10, 0.19)	0.17 (0.11, 0.23)	0.20 (0.13, 0.26)

Supplementary Table 4: Potential floodplain extent, population and assets to 100-year extreme coastal flood event in 2050 by RSLR Assumptions. The median and, in parentheses, the maximum and minimum values are provided.

RSLR Assumptions	Areas under 100-year (1000 km^2)	People under 100-year (million)	Asset under 100-year (US$ billion)
1) Climate-induced SLR only	53 (52, 55)	35 (34, 37)	5263 (3824, 7035)
2) SLR+GIA+Tectonics	53 (51, 55)	35 (34, 37)	5235 (3803, 6991)
3) SLR+GIA+Tectonics+Delta	54 (53, 56)	36 (34, 38)	5351 (3889, 7131)
4) SLR+GIA+Tectonics+Delta+City (min)	64 (62, 65)	41 (39, 43)	6215 (4460, 8146)
5) SLR+GIA+Tectonics+Delta+City (max)	74 (72, 76)	48 (46, 50)	7336 (5286, 9566)
6) SLR+GIA+Tectonics+Delta+City (control)	57 (56, 60)	37 (36, 39)	5609 (4083, 7459)

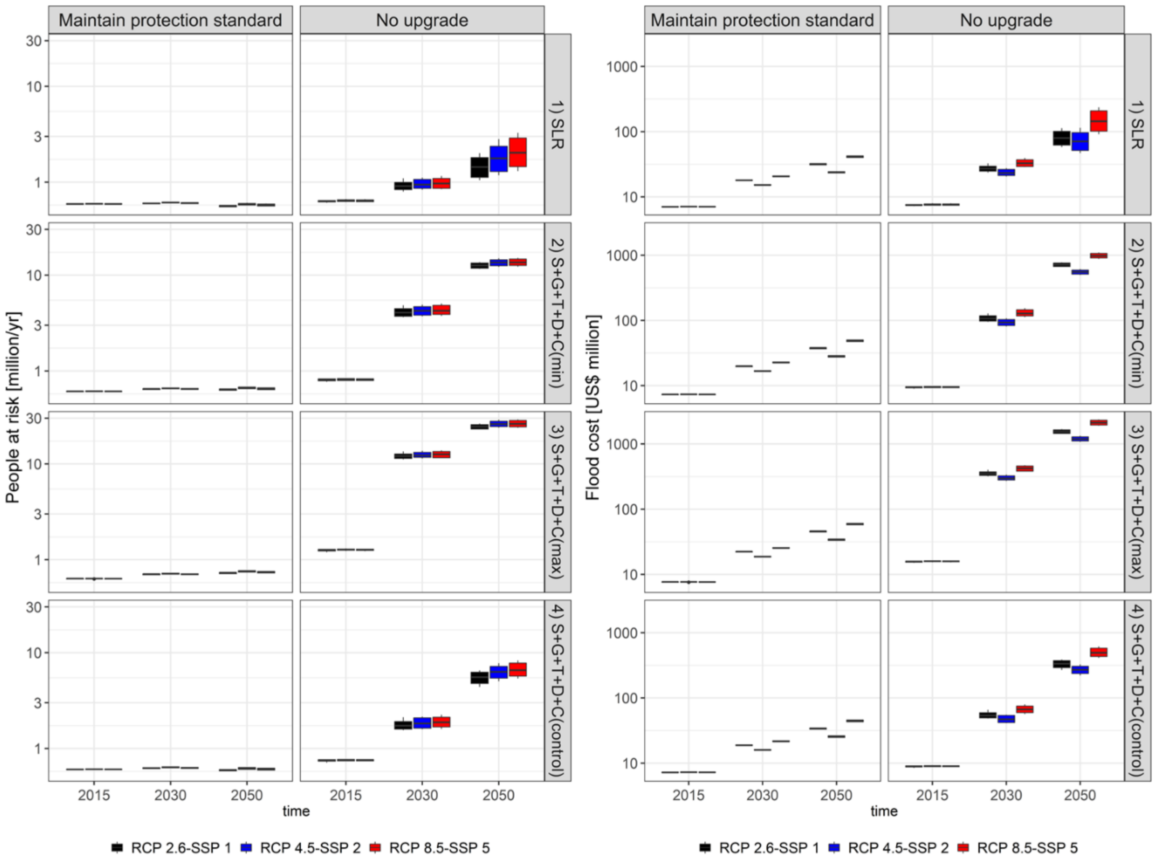

Supplementary Fig. 5: People at risk annually and annual flood costs caused by coastal flooding under maintain protection standard and no upgrade to protection strategy. On each box, the central mark indicates the median, and the bottom and top edges of the box indicate the 25th and 75th percentiles, respectively. The whiskers extend to the most extreme (maximum and minimum) data points. 1) Climate-induced SLR only; 2) Climate-induced SLR, GIA, tectonic movement, delta and uncontrolled minimum city subsidence; 3) Climate-induced SLR, GIA, tectonic movement, delta and uncontrolled maximum city subsidence; 4) Climate-induced SLR, GIA, tectonic movement, delta and controlled city subsidence.

Data 1 (separate file).

This file contains the observational subsidence values, measurements for subsidence records of coastal China basin obtained through a literature review.

The source data and the R code used to produce the numbers, tables and figures are available from https://doi.org/10.5281/zenodo.6969115 (20).

Supplementary References

1 Hinkel, J. *et al.* Coastal flood damage and adaptation costs under 21st century sea-level rise. *P Natl Acad Sci USA* **111**, 3292-3297, doi:10.1073/pnas.1222469111 (2014).

2 Nicholls, R. J. *et al.* A global analysis of subsidence, relative sea-level change and coastal flood exposure. *Nature Climate Change* **11**, 338-342, doi:10.1038/s41558-021-00993-z (2021).

3 Fang, J. Y. *et al.* Coastal flood risks in China through the 21st century - An application of DIVA. *Science of the Total Environment* **704**, 135311 (2020).

4 Peltier, W. R., Argus, D. F. & Drummond, R. Space geodesy constrains ice age terminal deglaciation: The global ICE-6G_C (VM5a) model. *J Geophys Res-Sol Ea* **120**, 450-487, doi:10.1002/2014jb011176 (2015).

5 Li, C.X. & Feng, Y. The characteristics of Chinese coast and relative sea level rising. In: *The influence of sea level rise on delta area of China and its countermeasures*. Division of Earth Sciences, Chinese Academy of Sciences. (1994). (in Chinese)

6 Lu, Y. & Ding. G. Neotectonic movements in the coastal zones of China. In: *The influence of sea level rise on delta area of China and its countermeasures*. Division of Earth Sciences, Chinese Academy of Sciences. (1994). (in Chinese)

7 Feng, H. et al. The research on the earth crust vertical movement characteristic and mechanism in Eastern China. *Acta Geodaetica et Cartographica Sinica* **27**, 16- 23 (1998). (in Chinese)

8 Cai, F., Su, X. Z., Liu, J. H., Li, B. & Lei, G. Coastal erosion in China under the condition of global climate change and measures for its prevention. *Prog Nat Sci-Mater* **19**, 415-426, doi:10.1016/j.pnsc.2008.05.034 (2009).

9 Syvitski, J. P. M. *et al.* Sinking deltas due to human activities. *Nat Geosci* **2**, 681-686, doi:10.1038/Ngeo629 (2009).

10 Ren, M. Relative sea level in Huanghe, Changjiang and Zhujiang (Yellow, Yangtze and Pearl River) delta over the last 30 years and predication for the next 40 years (2030). *Acta Geographica Sinica* 48(05), 385-393 (1993). (in Chinese)

11 Sun, Q. et al. Potential impacts of sea level rise on the economy and environment in the Yangtze River Delta and the Countermeasures thereof. *Resources and Environment in the Yangtze Valley* **6**, 59-65. (1997). (in Chinese)

12 Liu, D. Relative Possible impacts of relative sea level rise in the coastal areas in China. *Marine Forecasts* **2**, 21-28 (2004). (in Chinese)

13 Meckel, T. A., Ten Brink, U. S. & Williams, S. J. Sediment compaction rates and subsidence in deltaic plains: numerical constraints and stratigraphic influences. *Basin Res* **19**, 19-31, doi:10.1111/j.1365-2117.2006.00310.x (2007).

14 Ericson, J. P., Vorosmarty, C. J., Dingman, S. L., Ward, L. G. & Meybeck, M. Effective sea-level rise and deltas: Causes of change and human dimension implications. *Global Planet Change* **50**, 63-82, doi:10.1016/j.gloplacha.2005.07.004 (2006).

15 Shi, C. X. *et al.* Land subsidence as a result of sediment consolidation in the Yellow River delta. *J Coastal Res* **23**, 173-181, doi:10.2112/39951.1 (2007).

16 Tan, J. Y. et al. Estimation of sediment compaction and its contribution to land subsidence

in the Yellow River Delta. *Mar Geol Quat Geol* **31**, 33–38 (2014). (in Chinese)

17 Zhang, Y. et al. Self-weight consolidation and compaction of sediment in the Yellow River Delta, China. *Physical Geography* **1**, 1–15. doi:10.1080/02723646.2017.1347420 (2018).

18 Zhang, Y. et al. Spatial and temporal variations in subsidence due to the natural consolidation and compaction of sediment in the Yellow River delta, China. *Marine Georesources & Geotechnology* **37**, 152-163 (2019).

19 MLR (Ministry of Land and Resources) and MWR (Ministry of Water Resources). The prevention and control planning of land subsidence in China (2011-2020). (2012) (in Chinese)

20 Fang, J., Nicholls, R.J., Brown, S. et al. Benefits of subsidence control for coastal flooding in China [Data set]. Zenodo (2022). https://doi.org/10.5281/zenodo.6969115.
