# Supplementary material for: Benefits of subsidence control for coastal flooding in China
Source: Nat Commun. 2022 Nov 14;13:6946. doi: 10.1038/s41467-022-34525-w (PMC9663704; doi:10.1038/s41467-022-34525-w)
Supplement: Supplementary file 2 — Description of Additional Supplementary Files [file 41467_2022_34525_MOESM2_ESM.pdf]

Description of Additional Supplementary Files

File Name: Supplementary Data 1

Description: Contains the observational subsidence values, measurements for subsidence records of coastal China basin obtained through a literature review.
